# Contribution of Common Genetic Variants to Risk of Early-Onset Ischemic Stroke

**DOI:** 10.1212/WNL.0000000000201006

**Published:** 2022-10-18

**Authors:** Thomas Jaworek, Huichun Xu, Brady J. Gaynor, John W. Cole, Kristiina Rannikmae, Tara M. Stanne, Liisa Tomppo, Vida Abedi, Philippe Amouyel, Nicole D. Armstrong, John Attia, Steven Bell, Oscar R. Benavente, Giorgio B. Boncoraglio, Adam Butterworth, for the Cervical Artery Dissections and Ischemic Stroke Patients (CADSIP) Consortium, Jara Carcel-Marquez, Zhengming Chen, Michael Chong, Carlos Cruchaga, Mary Cushman, John Danesh, Stéphanie Debette, David J. Duggan, Jon Peter Durda, Gunnar Engstrom, Chris Enzinger, Jessica D. Faul, Natalie S. Fecteau, Israel Fernandez-Cadenas, Christian Gieger, Anne-Katrin Giese, Raji P. Grewal, Ulrike Grittner, Aki S. Havulinna, Laura Heitsch, Marc C. Hochberg, Elizabeth Holliday, Jie Hu, Andreea Ilinca, for the INVENT Consortium, Marguerite R. Irvin, Rebecca D. Jackson, Mina A. Jacob, Raquel Rabionet, Jordi Jimenez-Conde, Julie A. Johnson, Yoichiro Kamatani, Sharon L.R. Kardia, Masaru Koido, Michiaki Kubo, Leslie Lange, Jin-Moo Lee, Robin Lemmens, Christopher R. Levi, Jiang Li, Liming Li, Kuang Lin, Haley Lopez, Sothear Luke, Jane Maguire, Patrick F. McArdle, Caitrin W. McDonough, James F. Meschia, Tiina Metso, Martina Müller-Nurasyid, Timothy D. O'Connor, Martin O'Donnell, Leema R. Peddareddygari, Joanna Pera, James A. Perry, Annette Peters, Jukka Putaala, Debashree Ray, Kathryn Rexrode, Marta Ribases, Jonathan Rosand, Peter M. Rothwell, Tatjana Rundek, Kathleen A. Ryan, Ralph L. Sacco, Veikko Salomaa, Cristina Sanchez-Mora, Reinhold Schmidt, Pankaj Sharma, Agnieszka Slowik, Jennifer A. Smith, Nicholas L. Smith, Sylvia Wassertheil-Smoller, Martin Söderholm, O. Colin Stine, Daniel Strbian, Cathie L.M. Sudlow, Turgut Tatlisumak, Chikashi Terao, Vincent Thijs, Nuria P. Torres-Aguila, David-Alexandre Trégouët, Anil M. Tuladhar, Jan H. Veldink, Robin G. Walters, David R. Weir, Daniel Woo, Bradford B. Worrall, Charles C. Hong, Owen A. Ross, Ramin Zand, Frank-Erik de Leeuw, Arne G. Lindgren, Guillaume Pare, Christopher D. Anderson, Hugh S. Markus, Christina Jern, Rainer Malik, Martin Dichgans, Braxton D. Mitchell, Steven J. Kittner, the Early Onset Stroke Genetics Consortium of the International Stroke Genetics Consortium (ISGC)

**Affiliations:** From the Division of Endocrinology (T.J., H.X., B.J.G, B.D.M., K.A.R., J.A.P., P.F.M.), Diabetes and Nutrition, Department of Neurology (J.W.C., N.S.F., H.L., S.J.K.), Division of Rheumatology and Clinical Immunology (M.C.H.), Department of Medicine, Department of Epidemiology and Public Health (M.C.H.), and Institute for Genome Sciences (T.D.O.C.), University of Maryland School of Medicine; VA Maryland Health Care System (J.W.C.); Centre for Medical Informatics (K.R., C.L.M.S.), Usher Institute, University of Edinburgh, United Kingdom; Institute of Biomedicine (T.M.S., C.J.), Department of Laboratory Medicine, Sahlgrenska Academy, University of Gothenburg, Sweden; Department of Neurology (L.T., J.P., D.S., T.T.), Helsinki University Hospital and University of Helsinki, Finland; Department of Molecular and Functional Genomics (V.A., J.L., R.Z.), Geisinger Health System, Danville, PA; LabEx DISTALZ-U1167 (P.A.), RID-AGE-Risk Factors and Molecular Determinants of Aging-Related Diseases, University of Lille; Inserm U1167 (P.A.), Lille; Centre Hospitalier Universitaire Lille (P.A.); Institut Pasteur de Lille (P.A.), France; Department of Epidemiology (N.D.A., M.R.I.), University of Alabama at Birmingham; School of Medicine and Public Health (J.A., E.H.), University of Newcastle and Hunter Medical Research Institute, Australia; Stroke Research Group (S.B., H.S.M.), Department of Clinical Neurosciences, British Heart Foundation Cardiovascular Epidemiology Unit (A.B., J.D.), Department of Public Health and Primary Care, British Heart Foundation Centre of Research Excellence (A.B., J.D.), National Institute for Health Research Blood and Transplant Research Unit in Donor Health and Genomics (A.B., J.D.), University of Cambridge (A.B., J.D.), United Kingdom; Department of Neurology (Q.R.B.), University of British Columbia, Vancouver, Canada; Department of Cerebrovascular Diseases (G.B.B.), Fondazione IRCCS Istituto Neurologico “Carlo Besta,” Milan, Italy; Health Data Research UK Cambridge (A.B., J.D.); Wellcome Genome Campus (A.B., J.D.), Cambridge, United Kingdom; Stroke Pharmacogenomics and Genetics group (J.C.-M., I.F.-C., N.P.T.-A.), Biomedical Research Institute Sant Pau (IIB Sant Pau), Barcelona, Spain; MRC Population Health Research Unit (Z.C., R.G.W.), Nuffield Department of Population Health, University of Oxford, United Kingdom; Nuffield Department of Clinical Neurosciences (P.M.R.), University of Oxford, United Kingdom; DBCVS Research Institute (M.C., G.P.), Department of Pathology and Molecular Medicine, Population Health Research Institute, McMaster University; Thrombosis & Atherosclerosis Research Institute (TaARI) (M.C., G.P.), Hamilton, Ontario, Canada; Departments of Neurology (J.-M.L.) and Psychiatry (C.C.), Washington University School of Medicine, St. Louis, MO; Department of Medicine and Laboratory for Clinical Biochemistry Research (J.P.D.), Department of Medicine, (M.C.), University of Vermont Larner College of Medicine, Burlington, VT; Department of Human Genetics (J.D.), Wellcome Sanger Institute, Hinxton, United Kingdom; University of Bordeaux (S.D., D.-A.T.), Inserm, Bordeux Population Health Research Center, UMR 1219; Department of Neurology (S.D.), Institute for Neurodegenerative Disease, Bordeaux University Hospital, France; Quantitative Medicine and Systems Biology Division (D.J.D.), Translational Genomics Research Institute, An Affiliate of City of Hope, Phoenix, AZ; Laboratory for Clinical Biochemistry Research (J.P.D.), Department of Clinical Sciences (G.E., J.A.S., M.S., D.R.W.), Malmö and Department of Clinical Sciences (A.I., M.S., A.G.L.), Neurology, Lund, Lund University, Sweden; Department of Neurology (C.E., R.S.), Medical University Graz, Austria; Survey Research Center (J.D.F.), Institute for Social Research, University of Michigan, Ann Arbor; Stroke Pharmacogenomics and Genetics (I.F.-C.), Fundacio Docència i Recerca MutuaTerrassa, Spain; Unit of Molecular Epidemiology (C.G.), Institute of Epidemiology (C.G., A.P.), Helmholtz Zentrum München German Research Center for Environmental Health, Neuherberg; Klinik und Poliklinik für Neurologie (A.-K.G.), Kopf- und Neurozentrum, Universitätsklinikum Hamburg-Eppendorf, Germany; Neuroscience Institute (R.P.G., L.R.P.), Saint Francis Medical Center, Trenton, NJ; Department for Biostatistics and Clinical Epidemiology (U.G., ), Charité-University Medical Centre, Berlin, Germany; National Institute for Health and Welfare (A.S.H., V.S.), Helsinki, Finland; Departments of Emergency Medicine and Neurology (L.H.), Washington University School of Medicine, St. Louis, MO; Division of Women's Health (K.R.), Department of Medicine and Department of Neurology (C.D.A.), Brigham and Women's Hospital, Harvard Medical School; Department of Epidemiology (J.H.), Harvard T.H. Chan School of Public Health, Boston, MA; Department of Neurology and Rehabilitation Medicine (A.I.), Skane University Hospital, Lund, Sweden; Division of Endocrinology (R.D.J.), Diabetes and Metabolism, Department of Internal Medicine and the Center for Clinical and Translational Science, The Ohio State University, Columbus; Department of Neurology (M.A.J., A.M.T., F.E.d.L.), Radboud University Medical Center, Donders Medical Center for Neuroscience, Nijmegen, the Netherlands; Department of Genetics, Microbiology and Statistics (R.R.J.), Institute of Biomedicine (IBUB), University of Barcelona; Institut de Recerca Sant Joan de Déu (R.R.J.), Esplugues de Llobregat; Centro de investigación biomédica en red (CIBERER) (R.R.J.); Neurovascular Research Group (NEUVAS) (J.J.-C.), Neurology Department, Institut Hospital del Mar d’Investigacio Medica, Universitat Autonoma de Barcelona, Spain; Department of Pharmacotherapy and Translational Research and Center for Pharmacogenomics (J.A.J., C.W.M.), University of Florida, College of Pharmacy; Division of Cardiovascular Medicine (J.A.J.), College of Medicine, University of Florida, Gainesville; Laboratory of Complex Trait Genomics (Y.K.), Graduate School of Frontier Sciences and Department of Cancer Biology (M.K.), Institute of Medical Science, The University of Tokyo, Japan; Department of Epidemiology (S.L.R.K.), School of Public Health, University of Michigan, Ann Arbor; Department of Cancer Biology (M.K.), RIKEN Center for Integrative Medical Sciences (M.K., C.T.), Yokohama, Japan; Department of Medicine (L.L.), University of Colorado Denver, Anschutz Medical Campus, Aurora, CO; Department of Neurosciences, Experimental Neurology (R.L.), VIB Center, For Brain & Disease Research, KU Leuven–University of Leuven; Department of Neurology (R.L.), University Hospitals Leuven, Belgium; John Hunter Hospital (C.R.L.), Hunter Medical Research Institute and University of Newcastle, Newcastle, Australia and Priority Research Centre for Stroke & Brain Injury, University of Newcastle, NSW, Australia; Peking University Health Science Center (L.L.), Department of Epidemiology and Biostatistics, Peking University, Beijing, China; Department of Neurology (S.L., J.F.M., O.A.R.), Mayo Clinic, Jacksonville, FL; Faculty of Health (J.M.), School of Nursing and Midwifery, University of Technology Sydney, NSW, Australia; Department of Neurology (T.M.), Helsinki University Central Hospital, Helsinki, Finland; Institute of Genetic Epidemiology (M.M.-N.), Helmholtz Zentrum München–German Research Center for Environmental Health, Neuherberg; Institute of Medical Biostatistics, Epidemiology and Informatics (IMBEI), University Medical Center, Johannes Gutenberg University Mainz, Germany; Department of Medicine I, Ludwig-Maximilians University Munich, Germany; Department of Medicine (C.C.H.) University of Maryland School of Medicine, Baltimore, MD; Health Research Board Clinical Research Facility (M.O.D.), Geata an Eolais, National University of Ireland, Galway; Department of Neurology (J.P., A.S.), Jagiellonian University, Krakow, Poland; Institute for Medical Information Sciences (A.P.), Biometry and Epidemiology, Ludwig-Maximilians-University, Munich, Germany; Department of Epidemiology (D.R.), Bloomberg School of Public Health, Johns Hopkins University, Baltimore, MD; Psychiatric Genetics Unit (M.R., C.S.-M.), Group of Psychiatry, Mental Health and Addiction, Vall d'Hebron Research Institute (VHIR), Universitat Autònoma de Barcelona; Department of Psychiatry (C.S.-M.), Hospital Universitari Vall d'Hebron, Barcelona; Biomedical Network Research Centre on Mental Health (CIBERSAM) (M.R.), Instituto de Salud Carlos III, Madrid; Department of Genetics (M.R.), Microbiology, and Statistics, Faculty of Biology, Universitat de Barcelona, Spain; McCance Center for Brain Health (J.R., C.D.A.), Massachusetts General Hospital; Center for Genomic Medicine (J.R.), MGH; Department of Neurology (J.R.), MGH, Boston; Program in Medical and Population Genetics (J.R.), Broad Institute, Cambridge, MA; Department of Neurology and Evelin F. McKnight Brain Institute (T.R., R.L.S.), Miller School of Medicine, University of Miami, FL; Institute of Cardiovascular Research (P.S.), Royal Holloway University of London, and Ashford and St. Peters Hospital (P.S.), Surrey, United Kingdom; Group Health Research Institute (N.L.S.), Group Health Cooperative; Department of Epidemiology (N.L.S.), University of Washington; Seattle Epidemiologic Research and Information Center (N.L.S.), VA Office of Research and Development, Seattle, WA; Department of Epidemiology and Population Health (S.W.-S.), Albert Einstein College of Medicine, New York; BHF Data Science Centre (C.L.S.), Health Data Research UK, London, United Kingdom; Department of Neurology (T.T.) and Department of Clinical Genetics and Genomics (C.J.), Region Vastra Gotaland, Sahlgrenska University Hospital; Department of Clinical Neuroscience (T.T.), Institute of Neurosciences and Physiology, Sahlgrenska Academy at University of Gothenburg, Sweden; Stroke Theme (V.T.), Florey Institute of Neuroscience and Mental Health, University of Melbourne; Department of Neurology (V.T.), Austin Health, Heidelberg, Victoria, Australia; Department of Neurology (J.H.V.), University Medical Center Utrecht Brain Center, Utrecht University, the Netherlands; Department of Neurology and Rehabilitation Medicine (D.W.), University of Cincinnati College of Medicine, OH; Departments of Neurology and Public Health Sciences (B.B.W.), University of Virginia School of Medicine, Charlottesville; Section of Neurology (A.G.L.), Skåne University Hospital, Lund, Sweden; Program in Medical and Population Genetics (C.D.A.), Broad Institute of MIT and Harvard, Cambridge, MA; Institute for Stroke and Dementia Research (ISD) (R.M., M.D.), University Hospital, LMU Munich; Munich Cluster for Systems Neurology (SyNergy) (M.D.); German Center for Neurodegenerative Diseases (DZNE) (M.D.), Munich, Germany; Geriatric Research and Education Clinical Center (B.D.M., S.J.K.), Veterans Administration Medical Center, Baltimore, MD.

## Abstract

**Background and Objectives:**

Current genome-wide association studies of ischemic stroke have focused primarily on late-onset disease. As a complement to these studies, we sought to identify the contribution of common genetic variants to risk of early-onset ischemic stroke.

**Methods:**

We performed a meta-analysis of genome-wide association studies of early-onset stroke (EOS), ages 18–59 years, using individual-level data or summary statistics in 16,730 cases and 599,237 nonstroke controls obtained across 48 different studies. We further compared effect sizes at associated loci between EOS and late-onset stroke (LOS) and compared polygenic risk scores (PRS) for venous thromboembolism (VTE) between EOS and LOS.

**Results:**

We observed genome-wide significant associations of EOS with 2 variants in *ABO*, a known stroke locus. These variants tag blood subgroups O1 and A1, and the effect sizes of both variants were significantly larger in EOS compared with LOS. The odds ratio (OR) for rs529565, tagging O1, was 0.88 (95% confidence interval [CI]: 0.85–0.91) in EOS vs 0.96 (95% CI: 0.92–1.00) in LOS, and the OR for rs635634, tagging A1, was 1.16 (1.11–1.21) for EOS vs 1.05 (0.99–1.11) in LOS; *p*-values for interaction = 0.001 and 0.005, respectively. Using PRSs, we observed that greater genetic risk for VTE, another prothrombotic condition, was more strongly associated with EOS compared with LOS (*p* = 0.008).

**Discussion:**

The *ABO* locus, genetically predicted blood group A, and higher genetic propensity for venous thrombosis are more strongly associated with EOS than with LOS, supporting a stronger role of prothrombotic factors in EOS.

Substantial advances have been made in recent years toward identifying common genetic variation associated with risk of ischemic stroke.^1,2^ This progress has been largely based on meta-analyses of genome-wide association study (GWAS) results derived from predominantly late-onset cases. Given that a higher heritability of early-onset ischemic stroke is observed in multiple studies,^3-6^ there is a strong need for genetic studies focusing on early-onset stroke (EOS). A pressing question is whether the genetic contribution to EOS includes novel or specific mechanisms that may have translational importance across the whole age spectrum, as has been found from studies of early-onset cases in other complex diseases.^7-9^

Because atherosclerosis is a less common cause of stroke in young adults, we hypothesized that nonatherosclerotic, prothrombotic mechanisms may be more important and discernible in studies of EOS.^10,11^ This concept is supported by associations reported between EOS and multiple prothrombotic candidate genes.^10-14^ In this report, we present findings from the Genetics of Early Onset Ischemic Stroke Consortium, contrast the effect sizes of known stroke loci in early-onset vs late-onset stroke (LOS), and evaluate differing contributions of prothrombotic loci to EOS and LOS.

## Methods

tThe Early Onset Stroke Consortium (EOSC) is a collaboration of 48 different studies across North America, Europe, Japan, Pakistan, and Australia for a GWAS meta-analysis of early-onset ischemic stroke in cases aged 18–59 years. Collectively, these studies contributed 16,880 cases (16,730 cases included for analysis) and 601,413 nonstroke controls (599,237 included for analysis) (eMethods and eTable 1, links.lww.com/WNL/C245). All patients had brain imaging to exclude diagnoses other than ischemic stroke. Additional screening was performed in most studies to exclude cases believed to be due to a known monogenic cause (e.g., sickle cell disease) or nongenetic cause (e.g., drug use, complications of procedures). Ischemic stroke subtyping was performed using the Trial of Org 10 172 in Acute Stroke Treatment (TOAST) criteria^15^ by most sites.

The EOSC includes cases from 2 different sources: EOS cases who previously participated in the Stroke Genetics Network (SiGN)^16^ (n = 7,619) and EOS cases from additional non-SiGN study sites (n = 9,598). eTable 1 (links.lww.com/WNL/C245) lists the 48 sites contributing EOS cases and sources of controls. With 1 exception (Group 7 including cases from Barcelona and BASICMAR), controls from each study were of the same age or older than cases. Analysis groups were assigned as previously described to combine cases and controls of similar genetic ancestry groups and genotyped on arrays of similar density.^16^ Clinical characteristics of stroke cases are shown in eTable 2. Genotypes for all studies except Helsinki were imputed using the TOPMed reference panel on the University of Michigan Imputation Server.^17^ The Helsinki Study imputed genotypes using a Finnish population–specific reference panel and the BEAGLE software. All genotype data were based on genome build hg38. Genotyping platforms and cohort-specific quality control and analysis parameters are provided in eTable 3.

**Figure 1:**
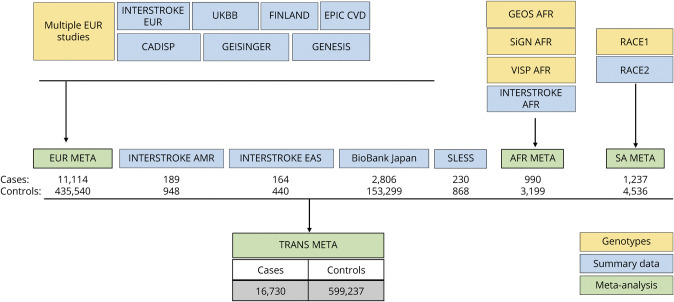
Overview of the Study Design

GWAS of stroke cases and controls were conducted within sites or within groupings of sites and then meta-analyzed. Before analysis, we removed cases if there were fewer than 40 in the analysis strata, if they could not be assigned to a genetic ancestry group, or if there was an inadequate number of controls. Filtering on these criteria left 16,730 (of 16,880) EOS cases for analysis. The overall analytic design is depicted in Figure 1. Our primary analysis was a transethnic meta-analysis. In parallel, we performed a European-only meta-analysis. Logistic regression was performed to test for association between stroke occurrence and single variants. Covariates included sex and up to 10 principal components to adjust for population stratification, unless otherwise specified. Power calculations indicated that our study provided 80% power to detect odds ratios (ORs) ranging from 1.09 to 1.20 for common genetic variants with minor allele frequencies (MAFs) > 5% at the genome-wide threshold for significance, that is, 5 × 10^−8^. For comparison, the previously detected ORs for ischemic stroke from MEGASTROKE GWAS (67,162 cases) ranged from 1.05 to 1.09.^1^ We also performed TOAST-defined stroke subtype analyses for sites providing subtype classification.

We compared effect sizes between EOS and LOS cases of European ancestry at 40 loci previously associated with stroke in MEGASTROKE,^1^ MEGASTROKE and UK Biobank combined,^2^ or in a previously published meta-analysis of small vessel stroke.^18^ We created an LOS case cohort (age onset ≥60 years) from the SiGN Consortium consisting of 9,272 LOS cases and 25,124 controls of European ancestry for this comparison. Effect sizes between EOS and LOS were compared using a Wald test.

To explore associations of serologically defined ABO blood groups (i.e., A, B, AB, and O) with stroke, we compared the distribution of blood groups among EOS, LOS, and controls. We assigned ABO blood groups using genotypes at 2 single-nucleotide polymorphisms (SNPs; rs8176719 and rs8176746), as described by Groot et al.^19^ (see eMethods, links.lww.com/WNL/C245).

ABO blood groups can be further subdivided into 5 different haplotypes, or subgroups, each of which can be tagged by a single SNP^20^ (see eMethods, links.lww.com/WNL/C245). The 5 *ABO* subgroups are A1 (the ancestral subgroup, tagged by rs2519093-T), O1 (tagged by a frameshift deletion, rs8176719-delG), A2 (tagged by rs1053878-A), B (tagged by rs8176743-T), and O2 (tagged by rs41302905-T).^20^ A study^20^ has recently shown that relative to subgroup O1, the genetically defined subgroups A1 and B are strongly associated with venous thrombosis risk, and subgroup A2 is associated with a modest increase in risk.

We hypothesized that the strong association of EOS with the *ABO* locus was related to the prothrombotic properties of the ABO blood group. We evaluated this hypothesis first by testing whether the 2 lead *ABO* variants were associated with venous thromboembolism (VTE), another prothrombotic condition, in the UKB and whether the association was more prominent in early-onset compared with later-onset VTE. Using summary-level association results (VTE results from the INVENT Consortium^21^), we estimated pairwise genetic correlations among EOS, LOS, and VTE using LD Score Regression analysis (LDSC).^22^ We then tested whether genetic predisposition to VTE, as measured by a polygenic risk score (PRS), was more prominently associated with EOS compared with LOS. For this purpose, we generated a VTE PRS for individuals from the EOSC and LOS subset from SiGN based on a large prior GWAS of VTE^23^ using PRSice software.^24^ The VTE PRS included 255 SNPs using a GWAS *p*-value threshold of 1 × 10^−5^ (see eMethods, links.lww.com/WNL/C245). We tested the association between the VTE PRS score with stroke in the European ancestry sample using logistic regression with 10 principal components for ancestry and sex included as covariates. Effect sizes between EOS and LOS stroke were compared using a Wald test (see eMethods).

We identified several disorders and plasma biomarkers (LOS, VTE, and plasma levels of von Willebrand factor [VWF] and factor VIII) for which associations at the *ABO* locus have previously been reported and then used the coloc software^25^ to assess evidence that the same causal SNPs associated with EOS also drove associations with the second trait. In brief, coloc uses a Bayesian approach and summary-level association results for 2 traits to calculate the posterior probabilities of 5 competing hypotheses (H0–H4) that assess whether the associations are due to the same (corresponding to H4) or a different (corresponding to H3) causal variant (see eMethods, links.lww.com/WNL/C245).

### Standard Protocol Approvals, Registrations, and Patient Consents

All participating sites obtained IRB or Ethics Board approval, and informed consent was obtained from all participants or their legally authorized representative.

### Data Availability

Summary results will be made available on application to the contact authors and consortium approval of the request. Individual-level data from a subset of sites will be made available on the database of Genotypes and Phenotypes (dbGaP).

## Results

### Genome-wide Analysis Results

**Figure 2:**
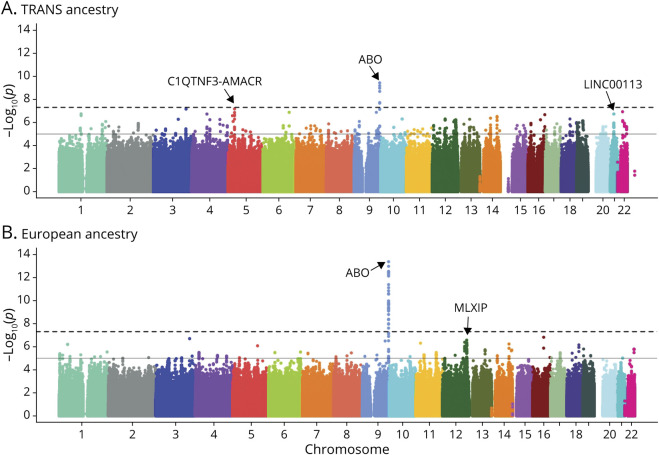
Genome-wide Association Analysis of EOS Manhattan plot of EOS meta-analysis in the EOSC, random effects model, in TRANS ancestry (A) and European Ancestry (B). EOS = early-onset stroke; EOSC = Early Onset Stroke Consortium.

The transethnic meta-analysis included a total of 16,730 EOS cases, representing 990 of African, 11,114 of European, 189 of Hispanic, 230 of Afro-Caribbean, and 4,207 of pan-Asian ancestry. There was little evidence for inflation of *p*-values across sites, as indicated by genomic control ranging from 0.86 to 1.14 (eTable 3, links.lww.com/WNL/C245). We identified 2 separate loci associated with EOS at genome-wide significance, both mapping to the *ABO* gene (Figure 2A and eTable 4). The lead variants at these loci tag ABO subgroups O1 and A1, respectively (see below). The OR for rs975381715, the O1-tagging variant (MAF = 0.37 in Europeans and 0.43 in African Americans), was 0.88 (95% confidence interval [95% CI]: 0.85–0.92; *p* = 3.72 × 10^−10^). The A1-tagging variant, rs8176685 (MAF = 0.19 in Europeans and 0.07 in African Americans), marks a 12 base-pair insertion/deletion polymorphism and had an OR of 1.16 (95% CI: 1.10–1.21; *p* = 2.06 × 10^−9^). This deletion variant has previously associated with ischemic stroke^1,26^ and is in near-perfect linkage disequilibrium (LD) with rs635634, the lead *ABO* SNP associated with all ischemic stroke in MEGASTROKE.^1^

The European ancestry-only meta-analysis, based on 11,114 EOS cases, also identified genome-wide associations marking the O1 and A1 blood groups the *ABO* gene, albeit with 2 different tagging SNPs (Figure 2B and eTable 5, links.lww.com/WNL/C245). In this analysis, the lead O1-tagging SNP was rs529565 (previously rs912805253) and had an OR = 0.88 (95% CI: 0.85–0.91; *p* = 4.31 × 10^−14^), and the lead A1-tagging SNP was rs635634 and had an OR = 1.16 (95% CI: 1.11–1.21; *p* = 6.54 × 10^−13^). Stratified analyses revealed no difference in effect sizes of either *ABO* rs529565 or rs635634 between men and women.

We performed a joint analysis of *ABO* SNPs rs529565 and rs635634 to assess their independent associations with EOS. The frequencies of the rs529565 T and rs635634 T alleles in European ancestry populations are 0.63 and 0.19, respectively. There was a moderate correlation between these 2 SNPs (r^2^ = 0.39), although the rs635634 T allele seems exclusively on the background of the rs529565 C allele, resulting in a D′ of 1 (eFigure 1, links.lww.com/WNL/C245). We therefore used a stratified approach to assess the contribution of rs529565 to stroke risk in the absence of the rs635634 T allele. In this analysis, restricted to strata for whom we had individual-level data, rs529565 remained associated with all EOS and at approximately the same effect size (OR = 0.90, 95% CI: 0.85–0.96; *p* = 0.003), implying an association of this SNP that was independent of rs635634.

In addition to the 2 genome-wide significant loci, we observed 1 additional SNP meeting genome-wide significance (rs118091666 in *SHKBP1*), although this SNP was rare (MAF = 0.018 in Europeans and monomorphic in non-Europeans). We observed 19 “suggestive” loci with subthreshold levels of significance (i.e., *p* < 1 × 10^−6^) in the transethnic analysis (eTable 4, links.lww.com/WNL/C245) and 14 loci in the European-only analysis (eTable 5). Among these loci were *MC4R* (melanocortin-4 receptor), an obesity-associated gene, and *TEK* (TEK receptor tyrosine kinase, rs78411354), which plays a role in embryonic vascular development. Three of the 29 unique loci associated with EOS in either the transethnic or European-only analysis at subgenome thresholds (i.e., *p* < 1 × 10^−6^) were nominally associated with LOS at *p* < 0.05 (eTables 4 and 5), and one of these, rs115133729 in *MAPKAPK5*, was strongly associated with LOS (*p* = 1.30 × 10^−7^) and has previously been associated with all ischemic stroke in MEGASTROKE Europeans (Cerebrovascular Disease Knowledge Portal, accessed April 16, 2022).

Analysis of stroke subtypes, available in 69.5% of all stroke cases, revealed 11 SNPs associated with various stroke subtypes at genome-wide thresholds of significance (eTable 6, links.lww.com/WNL/C245), although the sample sizes were relatively small for each subtype (ranging from 886 to 5,149 for transethnic analysis and 376 to 1,502 for European-only analysis) and the frequencies of the associated SNPs were low (8 SNPs with European ancestry (EUR) MAF <0.02 and MAF of the 3 SNPs <0.09).

There was no evidence for replication of the *HABP2* rs11196288 variant, which was previously associated with EOS in our earlier phase 1 transethnic meta-analysis from the EOSC,^12^ although its MAF is low (∼3% in gnomAD European ancestry populations). In the expanded meta-analysis presented in this report (16,927 currently vs 4,505 cases previously), there was no evidence for association of this SNP with EOS in any of the new sites, including those of non-European ancestry (eFigure 2, links.lww.com/WNL/C245).

### Associations of Index *ABO* Variants With All Stroke and Stroke Subtypes in EOS and LOS

As indicated above, the rs529565 T and rs635634 T alleles at the *ABO* locus tag blood subgroups O1 and AO1, respectively. The associations we observed for these SNPs with EOS are substantially higher than the peak associations previously reported at the *ABO* locus in predominantly older stroke populations (e.g., OR = 1.08; 95% CI: 1.05–1.11 in MEGASTROKE^1^). Both SNPs also had significantly larger effect sizes for all stroke in EOS compared with LOS (*ABO* rs529565: OR = 0.88 [95% CI: 0.85–0.91] vs 0.96 [95% CI: 0.91–1.00] and *ABO* rs635634: OR = 1.16 [95% CI: 1.11–1.21] vs 1.05 [95% CI: 1.00–1.10], *p*-values for interaction = 0.0004 and 0.001, respectively) (eFigure 3, links.lww.com/WNL/C245).

In stroke subtype–specific analyses, the O1-defining allele at rs529565 had higher effect sizes (i.e., was more protective) in EOS than in LOS for large artery stroke, cardioembolic stroke, and undetermined stroke (*p*-values for homogeneity = 0.026, 0.007 and 0.0004, respectively). Similarly, the effect sizes of the A1-defining SNP rs635634 were significantly higher in EOS than in LOS for cardioembolic stroke (*p* = 0.018) and for undetermined stroke *p* = 0.001) (eFigure 3, links.lww.com/WNL/C245).

We assessed the associations of *ABO* SNPs rs529565 (encoding blood subgroup O1) and rs2519093 (the defining SNP encoding blood subgroup A1 and in near-perfect LD with rs635634 [r^2^ = 0.99]) in the UKB as a quasireplication, “quasi” because EOS UKB cases were included as part of the primary EOSC analyses, but LOS cases were not. The analysis was limited to ischemic stroke cases and based on *ICD* codes, as described in the eMethods (links.lww.com/WNL/C245). Similar to our analysis in EOSC and SiGN, we observed stronger associations of both SNPs in EOS than in LOS. The ORs for *ABO* rs529565 (O1-defining) were 0.93 (95% CI: 0.86–0.99; *p* = 0.02) for EOS and 0.95 (95% CI: 0.90–0.99; *p* = 0.02) for LOS and for *ABO* rs2519093 (A1-defining) were 1.10 (95% CI: 1.01–1.19; *p* = 0.03) for EOS and 1.05 (95% CI: 1.00–1.02; *p* = 0.07) for LOS.

### Association of ABO Serologic Blood Group With EOS and LOS

We initially compared the distribution of A, B, AB, and O blood groups between EOS cases, LOS cases, and nonstroke controls. As indicated in Table 1, EOS cases were more likely to have blood group A and less likely to have blood group O compared with LOS cases and controls (*p* < 0.001 for each comparison). EOS and LOS cases were also more likely to have blood group B compared with controls (*p* = 0.004 and *p* = 0.012, respectively).

**Table 1:**
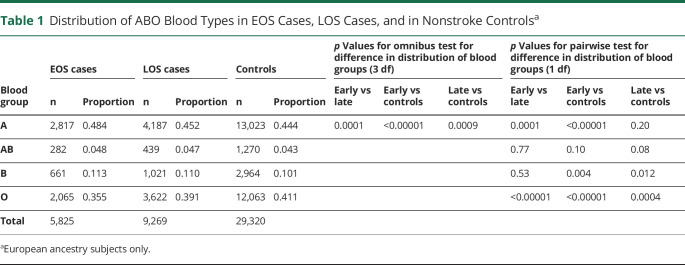
Distribution of ABO Blood Types in EOS Cases, LOS Cases, and in Nonstroke Controls^a^

### Associations of Genetically Defined ABO Blood Subgroup With EOS and LOS

We further compared associations of all 5 ABO blood subgroups between EOS and LOS cases of European ancestry. The top *ABO* SNPs identified in our GWAS, rs529565 and rs635634, are in high LD with the tagging SNPs for blood subgroups O1 and A1, which were not included in our data set (Table 2). We also used rs1137827 as a high LD tag for rs8176743 (blood subgroup B). As previously described and consistent with the VTE analysis of another study,^20^ we found blood subgroup O1 (rs529565) to be protective against EOS (OR = 0.88, 95% CI: 0.85–0.91; *p* = 4.31 × 10^−14^) and blood subgroup A1 (rs635634) to be strongly associated with EOS (OR = 1.16, 95% CI: 1.11–1.21; *p* = 6.54 × 10^−13^) (Table 2). Unlike for VTE, blood subgroup B (rs1137827) showed little evidence for association with EOS (OR = 1.05, 95% CI: 0.93–1.16; *p* = 0.324). These trends were also evident for LOS, although the strengths of association were markedly reduced, that is, blood subgroup A1 (OR = 1.05, 95% CI: 1.00–1.10, *p* = 0.044), and blood subgroup O1 (OR = 0.96, 95% CI: 0.92–1.00, *p* = 0.036).

**Table 2:**
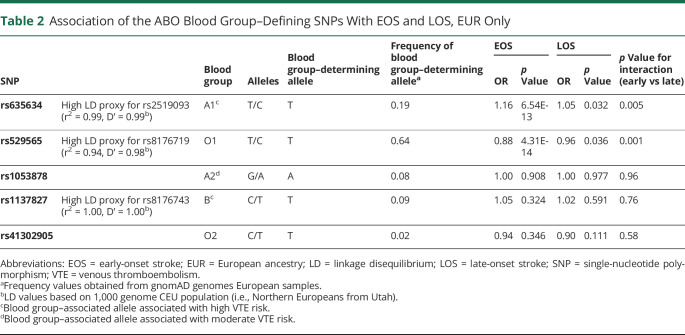
Association of the ABO Blood Group–Defining SNPs With EOS and LOS, EUR Only

Based on an allele frequency of 0.20 and OR of 1.16, we estimated that 6% of all EOS cases in Europeans can be attributed to rs635634, compared with ∼2% of LOS cases (see Supplement, eMethods, links.lww.com/WNL/C245).

To evaluate whether rs529565 and rs635634 accounted for all of the genetic effects at the *ABO* locus, we performed a conditional analysis in Europeans to test for association of all SNPs at this locus (±50 kb from *ABO*) with EOS after including rs529565 and rs635634 in the model as covariates. These analyses revealed 7 SNPs, falling within 4 LD groups, to be associated with all stroke at a *p*-value < 0.01. Of these, rs5598407 (MAF = 0.045) was the most strongly associated with stroke (OR = 1.21, *p* = 3.13 × 10^−4^). This SNP was in LD with all of the blood group–defining SNPs (r^2^ < 0.03 but D' = 1 for all) and with rs176694 (r^2^ = 0.347), which is strongly associated with E-selectin levels (*p* < 10^−406^) in the GWAS catalog.^27^ None of the tag SNPs for the 3 other blood subgroups (rs1053878-A2, rs1137827-B, and rs41302905-O2) showed evidence for association after conditioning on rs529565 (O1) and rs635634 (A1); *p* > 0.40 for all.

### Associations of Other Established Stroke Loci With EOS

We compared the effect sizes of 40 loci previously found to associate with ischemic stroke^1,2,16^ between EOS (age at first stroke: younger than 60 years) and LOS (60 years or older). As indicated in eTable 7 (links.lww.com/WNL/C245), the ORs associated with EOS were generally consistent with those estimated for LOS with 2 exceptions, *RGS7* rs146390073 and *TM4SF4* rs7610618, although the MAFs were relatively rare for both SNPs and there were no statistically significant differences between EOS and LOS.

### Genetically Defined ABO and Risk of Early-Onset and Late-Onset VTE

Because the *ABO* locus has been previously associated with VTE and other prothrombotic states,^28^ we assessed whether there was a similar graded age-at-onset association between *ABO* SNPs rs529565 (O1-defining) and rs2519093 (A1-defining) and VTE in the UKB. The *ABO* rs529565-O1 allele was more strongly associated with early-onset VTE (OR = 0.66, 95% CI: 0.62–0.69; *p* = 2.95 × 10^−58^) than with late-onset VTE (OR = 0.77, 95% CI: 0.74–0.81; *p* = 6.63 × 10^−28^); *p*-value for homogeneity of OR = 2.15 × 10^−6^). Similarly, the *ABO* rs2519093-A1 allele was more strongly associated with early-onset VTE (younger than 60 years; n = 3,514 cases) (OR = 1.64, 95% CI: 1.54–1.74; *p* = 1.42 × 10^−54^) than with late-onset VTE (60 years or older; n = 5,043 cases) (OR = 1.34, 95% CI: 1.27–1.41; *p* = 2.89 × 10^−29^); *p*-value for homogeneity of ORs = 4.01 × 10^−7^ (details in eMethods, links.lww.com/WNL/C245).

### Genetic Risk of VTE on Risk of EOS and LOS

We used 2 approaches to evaluate whether genetic risk of VTE confers risk for stroke and especially for earlier-onset stroke. First, using LDSC analysis on our current GWAS and the GWAS of VTE from INVENT, we estimated genetic correlations of 0.376 ± 0.153, *p* = 0.014, between VTE and EOS and 0.032 ± 0.198, *p* = 0.87, between VTE and LOS. The genetic correlation between EOS and LOS was 0.455 ± 0.128 (*p* = 0.0004).

Second, we measured genetic risk of VTE using a 255-SNP–based PRS,^23^ as described in the eMethods (links.lww.com/WNL/C245). A 1-SD unit increase in VTE PRS was associated with a 1.13-fold increase in risk of EOS (95% CI: 1.10–1.16, *p* < 5.21 × 10^−16^) and a 1.04-fold increase in risk of LOS (95% CI: 1.01–1.08, *p* = 0.010; *p*-value for homogeneity of OR = 0.0002). These results were essentially unchanged when the analysis was repeated after removing 7 SNPs at the *ABO* locus from the PRS.

### Colocation of Associations at the *ABO* Locus Between EOS and Other Thrombotic-Related Disorders and Biomarkers

**Figure 3:**
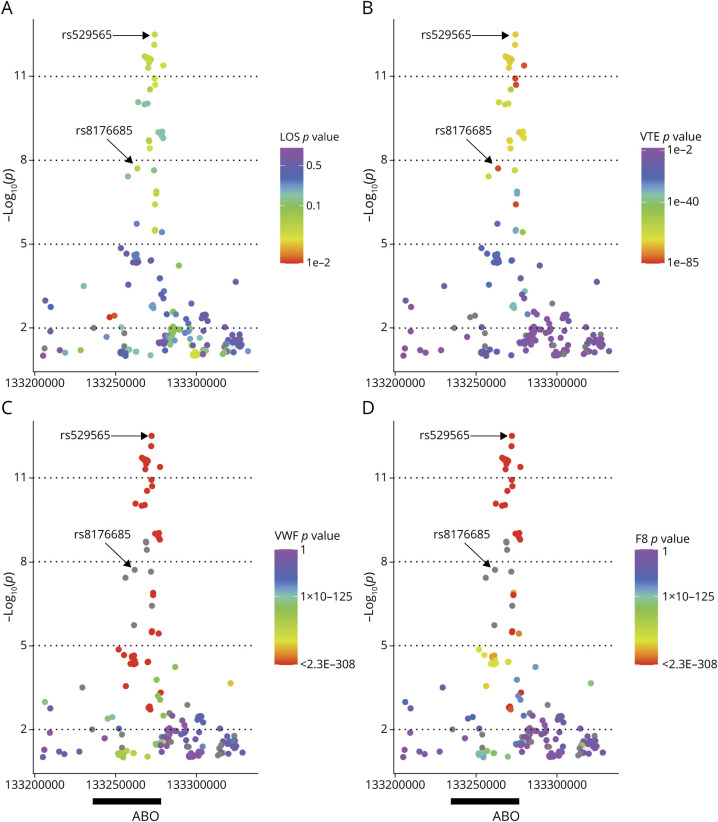
Associations of SNPs at the *ABO* Locus With Multiple Traits Association plots showing SNPs associated with EOS at the *ABO* locus (±50 kb) and the corresponding associations of these SNPs with LOS (A), VTE (B), plasma levels of VWF (C), and plasma levels of factor VIII (D). EOS = early-onset stroke; LOS = late-onset stroke; SNP = single-nucleotide polymorphism; VWF = von Willebrand factor.

Figure 3 provides a visualization of the SNPs most strongly associated with EOS and their corresponding associations with VTE and 2 prothrombotic biomarkers. Each panel shows the zoom plot depicting the association of SNPs at the *ABO* locus (±50 kb) with EOS. The heat map in each panel and the color coding of the variants depict the association with LOS (panel 3A), VTE (panel 3B), plasma levels of VWF (panel 3C), and plasma levels of factor VIII (F8, panel 3D). The SNPs most strongly associated with EOS tended also to be the ones most strongly associated with VTE, VWF, and F8. This trend was less apparent for LOS. Consistent with these observations, there was strong evidence to support the hypothesis of colocalization of at least 1 shared causal SNP between EOS and VTE, EOS and VWF, and EOS and FVIII (posterior probability supporting H4 > 99% for all pairs). Consistent with the weak and dispersed set of associations with LOS at this locus, there was insufficient evidence to strongly support either colocalization or absence of colocalization of shared causal SNPs between EOS and LOS (posterior probability supporting H4 = 39%; posterior probability supporting H3 = 61%).

## Discussion

Our analyses revealed 2 variants at the *ABO* locus that were highly associated with EOS. These variants tag 2 of the ABO blood subgroups, O1 and A1, showing a strong deleterious and protective association with ischemic stroke, respectively. Non-O blood groups have been associated previously with risk of ischemic stroke,^29-31^ but the novel contributions of our analysis are in showing a significantly stronger association of these blood groups with EOS compared with LOS and in linking risk predominantly to the blood subgroup A1. In particular, our analyses suggest that the ABO blood subgroups A1-tagging and O1-tagging variants (rs529565 and rs635634) are sufficient for capturing nearly all of the *ABO*-mediated genetic association with early-onset (and perhaps late) stroke. Stratified analyses indicate that both SNPs are independently associated with stroke, and further association analyses at the *ABO* locus that condition on the effects of these 2 SNPs reveal only modest additional signal at this locus.

Non-O blood groups have been associated with a variety of diseases and phenotypes, including arterial and venous thrombosis.^1,20,23,30,32,33^ The ABO blood groups are determined by the *ABO* gene, and the A and B allele encodes glycosyltransferase A and B, respectively, whereas the O allele encodes a nonactive enzyme. The glycosyltransferases add specific monosaccharides to the precursor H antigen, producing A and B antigens. These carbohydrate structures are expressed on red blood cells and on other cell types of importance for hemostasis, such as platelets and endothelial cells.^34^ These carbohydrates are also present on circulating solubilized glycoproteins, including VWF.^34^ It is well known that non-O blood groups have increased plasma levels of VWF and coagulation factor VIII,^20,34-36^ with the A1 subtype having the highest levels of both.^37^ The *ABO* locus has also been shown to associate with circulating levels of other glycoproteins such as tumor necrosis factor, soluble E-selection, P-selectin, intracellular adhesion molecule 1, and thrombomodulin.^38,39^

Because the *ABO* locus is so pleiotropic, several mechanisms may contribute to our finding of an association to EOS. However, taken together, our results clearly support an increased role of prothrombotic mechanisms in EOS compared with LOS. First, we have shown that the *ABO* rs529565-O1 SNP is not only associated with EOS but also more strongly associated with early-onset compared with late-onset VTE. Second, our results show that genetic risk of VTE, a well-recognized prothrombotic-related disorder, is also more strongly associated with EOS compared with LOS. Consistent with these observations, we further found that the EOS-associated haplotype colocalizes with deep venous thrombosis and with increased levels of VWF and FVIII, which are well-recognized prothrombotic factors.

Although our study had limited power to examine stroke subtypes, it is notable that the *ABO* O1 and A1-defining SNPs were also significantly associated with large artery atherosclerosis, cardioembolic, and undetermined stroke subtypes. This leads to the question, what are the clinical implications of an enrichment of prothrombotic mechanisms in EOS? Clinical translation will require a better understanding of the prothrombotic mechanisms in EOS and, likely, a personalized secondary prevention strategy. The effect sizes of the stroke-associated common variants at the *ABO* locus are too small per se to have immediate clinical implications, but gene-gene and gene-environment interaction deserve future study.^40^ One path to translation would be to identify gene-drug interactions (e.g., oral contraceptives and genetic risk for thrombosis) and determine whether the joint effect has implications for primary prevention. Additional research implications are that rare variant studies should target prothrombotic and related pathways, which could identify variants of larger effect size.

In addition to *ABO*, we detected genome-wide evidence for association of EOS with *SHKBP1* rs118091666. Given that the MAF of this SNP is very low and this locus has not previously been associated with stroke to our knowledge, further follow-up is warranted of this observation.

Our study is not without limitations. First, further fine-mapping and detailed functional experiments will be needed to identify the causal variants and detailed biological pathways that link *ABO* to increased risk of EOS. Second, although 35% of participants in the EOSC are of non-European ancestry, the diversity of the current EOSC cohort is still somewhat limited, reducing power to detect variants whose frequencies might be high in non-European populations yet low in Europeans. A third limitation is that the sample size even for all stroke is still small by GWAS standards; power to detect subtype-specific variants is even more limited.

In summary, our genome-wide analysis indicates a stronger association of *ABO* risk variants tagging blood groups O1 and A1 with EOS compared with LOS and stronger associations of the same *ABO* variants with early-onset compared with late-onset VTE, another prothrombotic condition. Similarly, we observed genetic risk for VTE to be more strongly correlated with EOS compared with LOS. Our findings are consistent with an increased role for prothrombotic mechanisms in EOS compared with LOS.

## Glossary

CI: confidence interval
EOS: early-onset stroke
EOSC: Early Onset Stroke Consortium
GWAS: genome-wide association study
*ICD-9*: *International Classification of Diseases–9*
LD: linkage disequilibrium
LDSC: LD Score Regression analysis
LOS: late-onset stroke
MAF: minor allele frequency
OR: odds ratio
PRS: polygenic risk score
SiGN: Stroke Genetics Network
VTE: venous thromboembolism
VWF: von Willebrand factor
